# 3D-Conformer of Tris[60]fullerenylated *cis*-Tris(diphenylamino-fluorene) as Photoswitchable Charge-Polarizer on GHz-Responsive Trilayered Core-Shell Dielectric Nanoparticles

**DOI:** 10.3390/molecules23081873

**Published:** 2018-07-27

**Authors:** He Yin, Min Wang, Tzuyang Yu, Loon-Seng Tan, Long Y. Chiang

**Affiliations:** 1 Department of Chemistry, University of Massachusetts Lowell, Lowell, MA 01854, USA; He_Yin@student.uml.edu (H.Y.); wangmin81@gmail.com (M.W.); 2 Department of Civil and Environmental Engineering, University of Massachusetts Lowell, Lowell, MA 01854, USA; Tzuyang_Yu@uml.edu; 3 Functional Materials Division, AFRL/RXA, Air Force Research Laboratory, Wright-Patterson Air Force Base, Dayton, OH 45433, USA; loon.tan@us.af.mil

**Keywords:** tri[60]fullerenylated *cis*-*cup*-tris(diphenylaminofluorene), 3D-configurated *cis*-*cup*-tris(C_60_-diphenylaminofluorene), trilayered core-shell gold-fullerosome nanoparticles, photoswitchable permittivity, amplification of relative dielectric constant

## Abstract

Novel 3D-configurated stereoisomers *cis*-*cup*-tris[C_60_>(DPAF-C_9_)] and *trans*-*chair*-tris[C_60_>(DPAF-C_9_)] were designed and synthesized in good yields. The former, with three C_60_> cages per molecule facing at the same side of the geometrical molecular cup-shape, was proposed to provide excellent binding interaction forces at the gold surface of core-shell *γ*-FeO_x_@AuNP nanoparticles and to direct the subsequent formation of a fullerene cage array (defined as *fullerosome*). Upon photoactivation of the Au-layer and *cis*-*cup*-tris[C_60_>(DPAF-C_9_)] itself, the degree of photoinduced intramolecular e^−^-transfer from DPAF to a C_60_> moiety was found to be largely enhanced by the accumulated plasmonic resonance energy at the near-field surface. Distribution of resulting negative charges along the outer (C_60_>)-derived fullerosome shell layer of the trilayered NPs was correlated with the detected photoswitchable dielectric amplification phenomena using white LED light at 1.0 GHz.

## 1. Introduction

**Figure 1:**
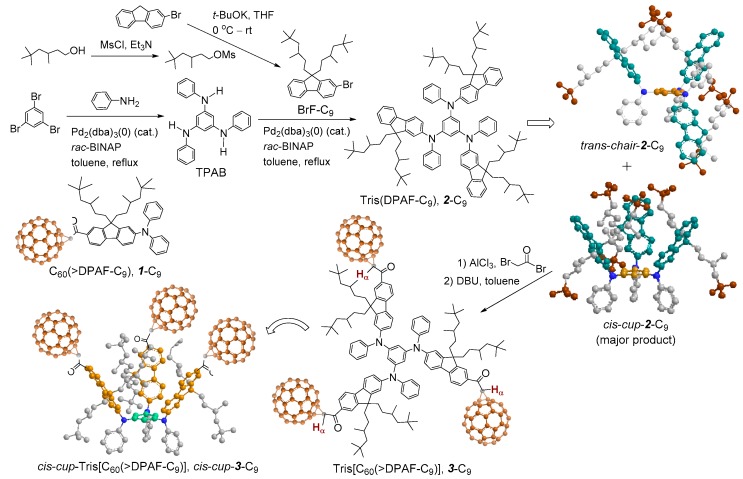
Synthetic route to the preparation of *cis*-*cup*-tris[C_60_(>DPAF-C_9_)] (*cis*-*cup*-**3**-C_9_) from the isolated precursor *cis*-*cup*-tris(DPAF-C_9_) (*cis*-*cup*-**2**-C_9_) with reaction reagents provided.

Electronic charge-polarization of organic substances under the externally applied field is the basic phenomenon leading to dielectric characteristics. It can be correlated with electrons shifting within polarizable molecules or the dielectric medium showing electronic polarizability. Certain polymers including poly(vinylidene fluoride) (PVDF, having asymmetrical electron-withdrawing fluorine) [1,2], polyarylene ether nitrile (PEN, having polar cyano groups) with conducting polyaniline [3,4] and azobenzene dyes (having large dipole moments of polar push-pull donor-acceptor terminal groups) [5] may exhibit such behavior; however, with a low dielectric constant. Advantages of organic dielectrics over inorganic ones, such as barium calcium titanate [6], boron nitride on graphenes [7], and titanium dioxide [8], lay on their facile chemical modification and easy processing, intrinsic excellent mechanical performance, recyclability, and multiple components blending. Example of the latter case was given by hybrid preparation of PEN on core-shell nanoparticles [9]. These dielectric materials and polymers may exhibit potential in applications of capacitor-type charge and energy storage [10,11,12] and organic photovoltaics [13]. Recently, we found [14,15,16] that the dielectric constant of a medium can be amplified to more than 200–350% by a photoswitching technique using the construction of multi-layered core-shell nanoparticles. They consist of two main photoresponsive components, such as organic electron (e^−^)-polarizable nanostructure of C_60_(>DPAF-C_9_) (**1**-C_9_, Figure 1) and inorganic plasmonic gold nanocrystal hybrids in a form of core and shell layered nanomaterials. The degree variation of dielectric amplification depends on the core-shell composition and the distribution of polarized charges.

The compound **1**-C_9_ analogous nanostructure is an electronically polarizable push-pull donor-acceptor type conjugative compound having a C_60_ cage as the acceptor moiety and a DPAF-C_9_ [9,9-di(3,5,5-trimethylhexyl)-2-diphenylaminofluorene] chromophore as the light harvesting donor antenna moiety. It is a part of the general composition of C_60_(>light-harvesting antenna)_x_ exhibiting ultrafast inter- and intramolecular photoinduced electron-transfer processes going from the electron-donating diphenylaminofluorene moiety to the electron-accepting C_60_ cage moiety [17,18,19,20]. The phenomena resulted in the formation of a positively charged (DPAF)^+^**·**-C_9_ and a negatively charged (C_60_>)^−^**·** cage moieties, respectively. These two charge states form the foundation of our observed photoswitchable dielectric property enhancements [14,15,16]. Recently, we further extended the study to design and synthesize highly restricted 3D-stereoisomers based on inter-connected three DPAF-C_9_ chromophore units giving a structure of tris(DPAF-C_9_) or **2**-C_9_ (Figure 1). The structural modification allowed us to investigate the stereo-configuration-dependence of organic fluorophore on the enhancement of their photophysical properties, including photoluminescence (PL) and electroluminescence (EL) emission in solid-state thin-films [21]. The stereochemical modification was based on the construction of 3D-geometrically branched chromophores having sterically hindered alkyl side-chains that resulted in the physical separation of each DPAF-C_9_ moiety of **2**-C_9_ from each other. It prevents and minimizes the tendency of planar organic *π*-conjugated fluorophore molecules, such as DPAF, to undergo aromatic-aromatic stacking, overlapping, and aggregation, via intermolecular hydrophobic-hydrophobic interactions, in solid state thin-films. The packing action triggers concentration-dependent self-quenching effects at excited states, resulted in the reduction of photophysical properties at the condensed phase. In the case of tris(DPAF-C_9_), attachment of large bulky groups at the vicinity of planar molecular benzene core region can restrict and reduce the degree of freedom in intramolecular DPAF-C_9_ rotation via steric hindrance, and thus the tendency of intermolecular aggregation. During the synthesis of tris(DPAF-C_9_), two stereoisomeric forms were isolated, namely, *trans*- and *cis*-tris(DPAF-C_9_) defined as *trans*-*chair*-**2**-C_9_ and *cis*-*cup*-**2**-C_9_ [22], respectively, as shown in Figure 1. We selected the latter *cup*-form to undergo further attachment of a C_60_> cage as the end group of each DPAF-C_9_ arm that resulted in a nanostructure of tris[60]fullerenylated *cis*-tris(diphenylaminofluorene) as *cis*-*cup*-tris[C_60_(>DPAF-C_9_)] or *cis*-*cup*-**3**-C_9_ for the photoswitchable dielectric property amplification study. This design of new stereo-isomeric structures may be beneficial for uses as positive charge carriers in enhancing photoinduced dielectric characteristics [23] and as the precursor building blocks in the synthesis of several C_60_- and C_70_-based ultrafast photoresponsive nanomaterials in correlation to our recent studies [17,18,19,20]. Therefore, we applied *cis*-*cup*-**3**-C_9_ as a photoswitchable dielectric charge-polarizer in the fabrication of microwave-responsive plasmonic trilayered core-shell nanoparticles.

## 2. Results and Discussion

An electromagnetic (EM) wave consists of both components of perpendicular electric-fields and magnetic-fields. Most organic photoresponsive materials are non-magnetic. Therefore, our main design consideration of organic microwave-responsive materials in tuning a material’s refractive index was based on the electric susceptibility, in terms of the degree of dielectric polarization in response to photoactivation instead of an applied electric field. In general, for disordered amorphous charge-transfer complex solids, the charge transfer events will not lead to significant alternation of the dielectric property of solids. Our approaches were to achieve the ferroelectric-like characteristics by firstly demonstrating ultrafast photoinduced intramolecular electron-transfer rate going from DPAF donor moiety to the C_60_> acceptor moiety of C_60_(>DPAF-C_9_) (**1**-C_9_) within <130 femtoseconds [17,18,19,20], resembling nearly spontaneous photoinduced charge-polarization action. Secondly, we then fabricated the C_60_> cage assembly into an aligned partial bilayered *fullerosome* membrane structure [24]. This type of fullerosome consists of a C_60_-C_60_ cage aligned array into a nano-layer to host the polarized negative charges at the inner membrane area and all DPAF donor-antennae moieties facing outside surface of the fullerosome to host the polarized positive charges. The self-assemble action was induced by strong hydrophobic-hydrophobic (C_60_>)-(C_60_>) interaction forces among fullerene cages. The resulting layered, polarized charges distribution affords a close resemblance to the characteristics of dielectrics, also as nano-capacitor-like assemblies, upon LED irradiation [14,15,16]. 

In the material’s design, we replaced **1**-C_9_ by *cis*-*cup*-tris[C_60_(>DPAF-C_9_)] (*cis*-*cup*-**3**-C_9_, Figure 1) as the outer shell donor-acceptor nanoconjugates on the construction of core-shell nanoparticles. It was derived from a *C*_3_-symmetrical 1,3,5-triaminobenzene ring as the central core for connecting three fused 2-diphenylaminofluorene moieties, forming a new class of 3D-stereomeric tris(fluorenylphenylamino)-benzene as tris(DPAF-C_9_) **2**-C_9_. These 3D-stereomers faces high torsional stress at the central benzene core region that forces all fluorene chromophore groups located at the outer-edge area to be oriented in a non-coplanar 3D-configuration [22]. Two stereoisomeric forms are possible, namely, *cis*-*cup*-**2**-C_9_ and *trans*-*chair*-**2**-C_9_ (Figure 1). They can be separated by thin-layer chromatographic technique with the former as the major product. Based on the density functional theory (DFT) calculation with the geometries optimized at the B97-D3/SVP level of theory [21], the *cis*-*cup*-form having three bis(3′,5′,5′-trimethylhexyl)fluorene moieties on 1,3,5-tris(phenylamino)benzene was found to be essentially *C_3_* symmetric with a higher formation tendency than the corresponding *trans*-*chair*-form. It is owing to the influence by strong dispersion interactions within the alkyl chains that are enhanceable in the presence of more polar media. Upon a 180^°^-rotation of one of three amino groups, the structure changes to produce a conformational isomer in a *trans*-*chair*-form which is less cup-like than the *cis*-*cup*-form. In principle, subsequent attachment of three C_60_> cages on *cis*-*cup*-**2**-C_9_ should lead to *cis*-*cup*-**3**-C_9_ having all C_60_> moieties facing outward from the central benzene core at the same side with each other. This will facilitate the cup-attachment on the gold layer of core-shell nanoparticles induced by the strong C_60_-Au binding force [25,26] and, thus, enhance the distribution of polarized negative charges at gold surface bound monolayered fullerosome region, as the positive contribution to the photoswitchable relative dielectric properties. 

### 2.1. Synthesis of Electron-Polarizable Tris[C_60_(>DPAF-C_9_)] Stereoisomer cis-cup-**3**-C_9_

**Figure 2:**
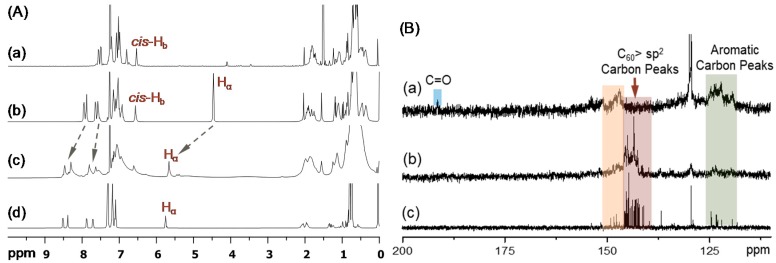
(**A**) ^1^H NMR spectra (CDCl_3_) of (a) *cis*-*cup*-tris(DPAF-C_9_) (*cis*-*cup*-**2**-C_9_), (b) *cis*-*cup*-tris(BrDPAF-C_9_), (c) *cis*-*cup*-tris[C_60_(>DPAF-C_9_)] (*cis*-*cup*-**3**-C_9_), and (d) C_60_(>DPAF-C_9_) as a reference for comparison. (**B**) ^13^C NMR spectra of (a) *cis*-*cup*-tris(BrDPAF-C_9_), (b) *cis*-*cup*-tris[C_60_(>DPAF-C_9_)], and (c) C_60_(>DPAF-C_9_) (**1**-C_9_), showing in (b) the main group of fullerenyl sp^2^ carbon peaks at *δ*140–147 that indicated the attachment of C_60_> cages on **2**-C_9_, as compared with those of (Bc).

Synthetic procedure for the preparation of tris(DPAF-C_9_) with the detailed spectroscopic characterization was reported previously by us [21,22]. Geometrically conformational isomer *cis*-*cup*-**2**-C_9_ was clearly isolated from the *trans*-*chair*-stereoisomer and confirmed by the use of high performance liquid chromatography (HPLC, µPorasil^™^ 125 Å, 10 µm, 35 × 300 mm, mobile phase: hexane-EtOAc (9.0:1.0, *v*/*v*), flow rate: 1.0 mL/min, detector: UV at *λ* 350 nm) in an analytical scale. For the scale-up separation, column chromatography (CC) using the same solvent system did not provide satisfactory results. However, the application of thin-layer chromatography (TLC) technique using the solvent eluent in slightly lower polarity (hexane-ethylacetate/9.5:0.5, *v*/*v*) than those of HPLC or the CC formed an overlap band of the stereoisomers *cis*-*cup*-**2**-C_9_ and *trans*-*chair*-**2**-C_9_. By cutting the corresponding fluorescent TLC band of tris(DPAF-C_9_) into two portions and performing several repeated TLC procedures, we were able to significantly increase the degree of product purity sufficiently enough to allow our confirmation of their entity by ^1^H NMR spectrum [21,22]. It indicated a higher *R*_f_ value for *cis*-*cup*-**2**-C_9_ than that of *trans*-*chair*-**2**-C_9_. With a *C*_3_ symmetry in structure, the former displayed a differentiable singlet peak for three central benzene protons (*cis*-H_b_) at *δ* 6.55 (Figure 2(Aa)), while the latter with a less symmetry resulted in two proton peaks (*trans*-H_b_) at *δ* 6.48 (1H) and 6.55 (2H). 

Prior to the attachment of three C_60_> cages on *cis*-*cup*-**2**-C_9_, it was functionalized by the Friedel–Crafts acylation at C7 position of diphenylaminofluorene moiety with *α*-bromoacetyl bromide in the presence of aluminum chloride (6.0 eq.) in 1,2-dichloroethane at 0 ^°^C to ambient temperature overnight to afford the corresponding *α*-bromoacetylfluorene derivative, as the intermediate step of reactions. It resulted in yellow semi-solids in 53% yield of *cis*-*N*^1^,*N*^3^,*N*^5^-tris(7-*α*-bromoacetyl-9,9-di(3′,5′,5′-trimethyl-1′-hexyl)fluoren-2-yl)-1″,3″,5″-tris(phenylamino)benzene as *cis*-*cup*-tris(BrDPAF-C_9_). It was purified by either column or TLC chromatography (silica gel, hexane–toluene, 3:2, *R*_f_ = 0.3 on TLC). The functional structure of *cis*-*cup*-tris(BrDPAF-C_9_) was verified by both infrared (FT-IR) and ^1^H NMR spectra with the former showing a strong carbonyl (-C=O) stretching absorption band centered at 1675 cm^−1^ indicating this functional group being linked on a phenyl moiety since a clear band absorption shift from 1725 cm^−1^, normally detectable for an alkyl carbonyl group, was evident. In the case of ^1^H NMR spectrum, tris(Br-DPAF-C_9_) displayed characteristic new peak signals of two methylene protons (H_α_) next to the carbonyl group of the *α*-bromoacetyl moiety at *δ* 4.46 (Figure 2(Ab)). Subsequence attachment of a C_60_> cage to each of three DPAF moieties was made via a cylopropylacetyl bridge by the treatment of three *α*-bromoacetylfluorene moieties of *cis*-*cup*-tris(BrDPAF-C_9_) with C_60_ in toluene in the presence of DBU at ambient temperature for 8.0 h. An excessive amount of C_60_ (4.5 eq.) was applied to avoid the formation of C_60_-bisadducts or trisadducts during the cyclopropanation reaction. The product of *cis*-*cup*-tris[C_60_(>DPAF-C_9_)] stereoisomer as *cis*-*cup*-**3**-C_9_ was confirmed by the evidence of changing solubility characteristics matching those of C_60_ and a slight shift of cyclopropyl keto group absorption in IR spectrum to *υ*_max_ 1627 cm^−1^, which was assigned to the carbonyl stretching band. It was also accompanied by two typical fullerenyl cage signals at *υ*_max_ 575 (w) and 529 (s) cm^−1^. These two bands are characteristic absorptions used to provide evidence of (C_60_>)-related monoadduct with relative intensity ratio differentiable from those of starting C_60_ itself. They will disappear from the spectrum once becoming a (C_60_>)-bisadduct. Therefore, this IR technique was applied often in the [60]fullerenyl product structure verification. In addition, disappearance of *α*-proton peaks of the α-bromoacetyl group at *δ* 4.46 (Figure 2(Ab)) with the appearance of new peaks centered at *δ* 5.67 (Figure 2(Ac)) can be indicative of successful formation of the cyclopropanyl keto-bridge between a C_60_> cage and the fluorene moiety, using previously reported C_60_(>DPAF-C_9_) characterization (Figure 2(Ad)) as the reference [17]. The latter peak *δ* 5.67 was assigned to the chemical shift of *α*-proton (H_α_) of **3**-C_9_ indicated in Figure 1. Its large down-fielded shift from the normal chemical shift value of a alkyl acetyl proton at ~*δ* 2.1–2.6 is due to the influence of strong [60] fullerenyl current in the close vicinity. Accordingly, the detection of *δ* 5.67 peak can also be served as the verification of short covalent-bonding between C_60_> and DPAF moieties. The half-height width of H_α_ peak in Figure 2(Ac) is slightly wider than that of Figure 2(Ab) that revealed less symmetrical environment among three H_α_ protons of *cis*-*cup*-**3**-C_9_. Similarly, by using ^13^C NMR spectrum of C_60_(>DPAF-C_9_) (Figure 2(Bc)) as the reference for comparison, we were able to detect calculated 29 fullerenyl sp^2^ carbon peaks corresponding to the C_60_> moiety (*C*_2_ symmetry) of *cis*-*cup*-tris[C_60_(>DPAF-C_9_)] as a matching multi-peak band in the range of *δ* 140–147 (Figure 2(Bb)). The reduced spectrum resolution is due to the increasing molecular complexity along with the reduced solubility of *cis*-*cup*-**3**-C_9_. 

### 2.2. Synthesis of cis-cup-Tris[C_60_(>DPAF-C_9_)]-Encapsulated Core-Shell Nanoparticles

**Figure 3:**
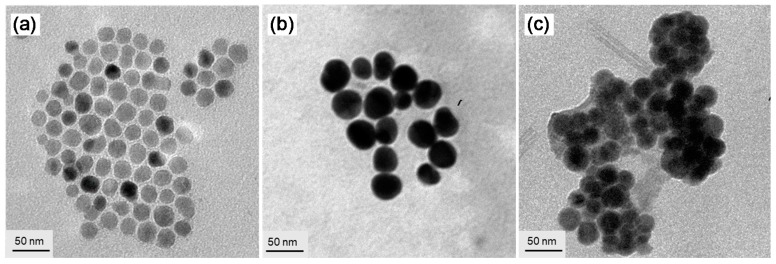
TEM micrograph images of (**a**) *γ*-FeO_x_ NPs, (**b**) *γ*-FeO_x_@AuNP (**4**-NPs), and (**c**) (*γ*-FeO_x_@AuNP)@{*cis*-*cup*-tris[C_60_(>DPAF-C_9_)]}_n_ (**5**-NPs), showing evolution of particle morphology changes and the soft organic material encapsulation on dark nanoparticles on the latter micrograph.

Synthetic procedures for the preparation of core-shell *γ*-FeO_x_@AuNP nanoparticles (**4**-NPs) in an average particle diameter of ~20 nm were reported by us recently [14,15]. A slightly modified method was applied in this study to afford similar results based on the verification by transmission electron microscopy (TEM) micrographs and microanalyses of energy dispersive x-ray spectra (EDS). Subsequent encapsulation of *γ*-FeO_x_@AuNPs by *cis*-*cup*-tris[C_60_(>DPAF-C_9_)] leading to the formation of trilayered core-shell NPs (Figure 3) was performed by mixing these two components in a predefined ratio in solvent under ultrasonication. The initial binding force of *cis*-*cup*-**3**-C_9_ to *γ*-FeO_x_@AuNP was governed by strong binding interaction forces of C_60_> cages to the gold surface [26,27] that should result in the partial replacement of 1-octanethiol capping molecules on the gold surface via alkylthiol ligand-C_60_> exchange induced by sonochemical energy to invert *cis*-*cup*-**3**-C_9_ molecules under ultrasonication. The exchange should form a monolayer of *cis*-*cup*-tris[C_60_(>DPAF-C_9_)] with the orientation of all three C_60_> cages per nanomolecule in a cup-capping manner facing on the surface of Au layer with interlinked three DPAF-C_9_ moieties facing outward. Attachment of additional *cis*-*cup*-**3**-C_9_ nanomolecules will follow the strong hydrophobic-hydrophobic interactions among (C_60_>)-(C_60_>) cages to form a partial trilayered fullerosome membrane resembling that of reported fullerenyl nanovesicle [24]. Since all encapsulated magnetic nanoparticles were physically removed from the container solution by the assistance of an external permanent magnet and washed repeatedly by ethanol and diethyl ether, we were able to ensure the products being free from residual non-binding *cis*-*cup*-**3**-C_9_ molecules at the NP surface. We assigned the resulting trilayered core-shell nanostructures of (*γ*-FeO_x_@AuNP)@{*cis*-*cup*-tris[C_60_(>DPAF-C_9_)]}_n_ as **5**-NPs.

TEM micrographs of Figure 3 showed the morphology and topography of NPs that indicated a roughly homogeneous narrow size distribution of the parent *γ*-FeO_x_ NPs (Figure 3a) at a diameter of ~20 nm, on average. Subsequent deposition of solid gold nanocrystals on the surface of *γ*-FeO_x_ NPs to a structure of core-shell *γ*-FeO_x_@AuNP (**4**-NP) was controlled to a shell layer thickness of 6.0–8.0 nm by the calculated quantity of HAuCl_4_ applied. This thickness was measured by the comparison of average particle size between Figure 3a,b, where the latter displayed much higher contrast of the Au layer shell. With the encapsulation of **4**-NPs by *cis*-*cup*-**3**-C_9_-derived fullerosome (array of C_60_> cages) as the outer shell layer, the morphology of soft organic materials in lighter contrast covering all hard nanoparticles can be observed that allowed us to measure an average thickness of roughly 10 nm (Figure 3c), matching well with the weight ratio of **4**-NPs and *cis*-*cup*-**3**-C_9_ applied in the core-shell nanoparticle fabrication. 

### 2.3. Physical Properties of cis-cup-Tris[C_60_(>DPAF-C_9_)] and Its Encapsulated Core-Shell Nanoparticles

**Figure 4:**
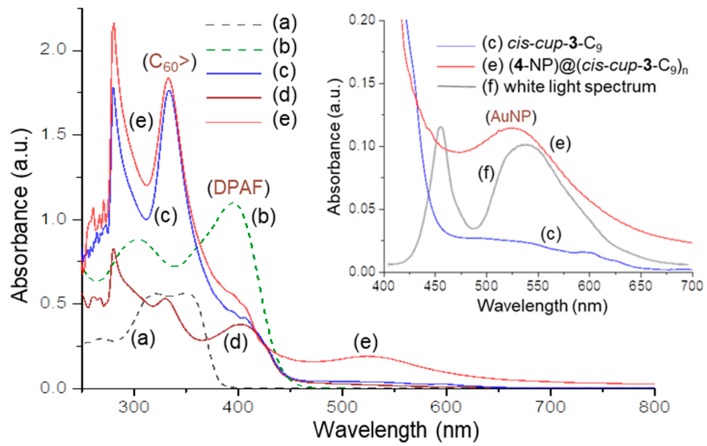
UV-vis spectra of (a) *cis*-*cup*-tris(DPAF-C_9_) (*cis*-*cup*-**2**-C_9_), (b) *cis*-*cup*-tris(BrDPAF-C_9_), (c) *cis*-*cup*-tris[C_60_(>DPAF-C_9_)] (*cis*-*cup*-**3**-C_9_), (d) C_60_(>DPAF-C_9_) (**1**-C_9_), (e) (**4**-NP)@(*cis*-*cup*-**3**-C_9_)_n_ (**5**-NPs), and (f) white light emission spectrum, where (a) and (b) were taken in EtOAc and (c), (d), and (e) were taken in toluene. The concentration of all samples is 1.0 × 10^−5^ M.

Photophysical properties of *cis*-*cup*-tris[C_60_(>DPAF-C_9_)] are dominated by two components, namely, C_60_> cages as the electron (e^−^)-acceptors and light-harvesting DPAF antenna units as electron (e^−^)-donors. Both components are photoresponsive at a different wavelength range. For example, optical absorption of C_60_> cages occurs mainly the band centered at 335 nm (1.76 × 10^5^ L mol^−1^ cm^−1^) [17], whereas the band centered at 402 nm (4.19 × 10^4^ L mol^−1^ cm^−1^) is attributed to the absorption of DPAF moieties, as shown in the UV–vis spectra of Figure 4c. Characteristics of the latter band were compared with that of *cis*-*cup*-**2**-C_9_ (Figure 4a), *cis*-*cup*-tris(BrDPAF-C_9_) (Figure 4b), and C_60_(>DPAF-C_9_) (**1**-C_9_, Figure 4d) showing a clear bathochromic shift of the 353-nm band of the former to 395 nm (1.10 × 10^5^ L mol^−1^ cm^−1^), which is matching roughly with the 404-nm band of **1**-C_9_ and the 402-nm band of *cis*-*cup*-**3**-C_9_ for the peak assignment. This assignment was also consistent with the observation of 2.9-folds higher in the absorption extinction coefficient (*ε*) value for *cis*-*cup*-tris(BrDPAF-C_9_), having three DPAF moieties per molecule, as comparing with that of **1**-C_9_ with one DPAF moiety per molecule. Upon the attachment of three C_60_> cages, optical absorptions of fullerene moieties of *cis*-*cup*-**3**-C_9_ became dominating in the spectrum with a much higher *ε* value for the 335-nm band (Figure 4c). It was accompanied by a weak characteristic (forbidden) steady state absorption band of the C_60_> moiety appearing at 692 nm (the insert of Figure 4c) that provided further confirmation of a fullerene conjugate structure.

Interestingly, distinctly 3.3-folds lower in the *ε* value of the 402 nm peak in Figure 4c than that of **1**-C_9_, making it as a shoulder band to the main C_60_> absorption, was interpreted by a phenomenon likely caused by the specific molecular 3D-conformation of *cis*-*cup*-tris[C_60_(>DPAF-C_9_)]. Owing to good solubility and compatibility of fullerenes to toluene solvent used, the orientation of three C_60_> cages was likely to face outward into the solvent phase while DPAF moieties were hidden inside the partially aggregated cluster nanoparticle. The hypothesis was also verified by observation of the distinguishable solubility decrease of *cis*-*cup*-**3**-C_9_ in CHCl_3_; however, increase in toluene and CS_2_ matches well with the solubility characteristics of C_60_. 

**Figure 5:**
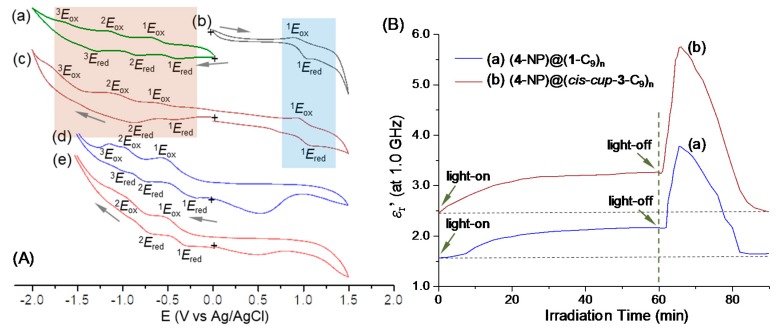
(**A**) Cyclic voltammograms (CV) of (a) C_60_>, (b) *cis*-*cup*-tris(DPAF-C_9_), (c) C_60_ (>DPAF-C_9_) (**1**-C_9_), (d) *cis*-*cup*-tris[C_60_(>DPAF-C_9_)] (with no light), and (e) *cis*-*cup*-tris[C_60_(>DPAF-C_9_)] (after white LED light irradiation for 30 min) at different voltages vs Ag/Ag^+^ in a solution concentration of 1.0 × 10^−3^ M in CH_3_CN-CH_2_Cl_2_, containing (*n*-butyl)_4_N^+^-PF_6_^−^ electrolyte (0.2 M), using Pt as working and counter electrodes and Ag/AgCl as the reference electrode at a scan rate of 10 mV/s. (**B**) Irradiation time-dependent relative dielectric constant (*ε*_r_′) amplification of trilayered core-shell nanoparticles of (a) (*γ*-FeO_x_@AuNP)@[C_60_(>DPAF-C_9_)]_n_ (**6**-NPs) and (b) (*γ*-FeO_x_@AuNP)@{*cis*-*cup*-tris[C_60_(>DPAF-C_9_)]}_n_ (**5**-NPs) at the frequency of 1.0 GHz. White LED light irradiation period was 60 min.

Our study of photoswitchable dielectric amplification phenomena is based on photoexcited plasmonic resonance energy transfer to induce the intramolecular charge polarization of *cis*-*cup*-tris[C_60_(>DPAF-C_9_)] forming the corresponding dielectric ion-radical components (C_60_>)^−^**·** and DPAF^+^**·**-C_9_. In this event, C_60_> served as an electron (e^−^)-acceptor, whereas light-harvesting DPAF antenna worked as an electron (e^−^)-donor. Accordingly, we first investigated the characteristics of redox potentials of both C_60_> and DPAF moieties in the 3D-configurated nanostructure. Measurements of the cyclic voltammetry (CV) were carried out on the sample of *cis*-*cup*-**3**-C_9_ in a mixture of toluene and (*n*-butyl)_4_N^+^-PF_6_^−^ electrolyte, using Pt as both working and counter electrodes and Ag/AgCl as the reference electrode. To provide a clear data interpretation, CV characteristics of C_60_ (>DPAF-C_9_) were applied as the reference under variation of cyclic oxidation and reduction voltages vs. Ag/Ag^+^ from −2.0 to 1.5 V. It displayed one reversible oxidation (^1^*E*_ox_ of 0.95 V)-reduction (^1^*E*_red_ of 1.07 V) cycle wave at positive voltages and at least two reversible reduction (^1^*E*_red_ of −0.43 V and ^2^*E*_red_ of −0.88 V)-oxidation (^1^*E*_ox_ of −0.62 V and ^2^*E*_ox_ of −1.10 V) cycle waves at negative voltages (Figure 5(Ac)). These potential values were compared by those of a set of separated molecular components using a C_60_> monoadduct and *cis*-*cup*-tris(DPAF-C_9_) as models for confirmation, showing reversible ^1^*E*_red_/^1^*E*_ox_ of −0.40/−0.71 V, ^2^*E*_red_/^2^*E*_ox_ of −0.82/−1.14 V, and ^3^*E*_red_/^3^*E*_ox_ of −1.33/−1.62 V vs. Ag/Ag^+^ for the C_60_> monoadduct (Figure 5(Aa)) and one reversible cyclic wave of ^1^*E*_ox_/^1^*E*_red_ as 0.93/1.03 V for *cis*-*cup*-tris(DPAF-C_9_) (Figure 5(Ab)). Once the compound **1**-C_9_ was molecularly tripled to *cis*-*cup*-**3**-C_9_, the cyclic redox wave corresponding to DPAF moieties at positive voltages fully disappeared, as shown in Figure 5(Ad). However, two clearly reversible ^1^*E*_red_/^1^*E*_ox_ (−0.23/−0.55 V) and ^2^*E*_red_/^2^*E*_ox_ (−0.75/−0.99 V) with one less obvious cyclic wave of ^3^*E*_red_/^3^*E*_ox_ (−0.97/−1.15 V) vs. Ag/Ag^+^ corresponding to the redox characteristics of C_60_> moieties of *cis*-*cup*-**3**-C_9_ were observed. This revealed the occurrence of a partial one-electron oxidation process of e^−^-donor DPAF-C_9_ moiety in the structure of *cis*-*cup*-**3**-C_9_ in the presence of three C_60_> cages. This inhibited the further electron-oxidation event in a clear manner. Since C_60_> is capable of undergoing at least three reversible reduction/oxidation waves (Figure 5(Aa)), partial e^−^-transfer from DPAF may not give significant influence on its CV diagram, except the first and second reduction potentials being modified from −0.43 to −0.23 and −0.88 to −0.75 V vs. Ag/Ag^+^, respectively. 

Most importantly, we were able to apply the white LED light irradiation (30 min) concurrent with the CV measurement to obtain the insight of redox characteristics of *cis*-*cup*-**3**-C_9_ under similar photoexcitation conditions used in the dielectric property measurements of the same compound in a form of core-shell nanoparticles, as described below. As a result, Figure 5(Ae) displayed a similar cyclic wave profiles as those of Figure 5(Ad) showing two clearly reversible waves with ^1^*E*_red_/^1^*E*_ox_ (−0.32/−0.55 V) and ^2^*E*_red_/^2^*E*_ox_ (−0.70/−0.99 V) vs. Ag/Ag^+^ that was attributed to the redox characteristics of C_60_> moieties. No clear oxidation waves of DPAF moieties were detected that was indicative of an effective intramolecular e^−^-transfer from DPAF-C_9_ to C_60_> upon light irradiation to produce three ion-radical pairs per molecule as *cis*-*cup*-tris[C_60_^−^**·**(>DPAF-C_9_)^+^**·**]. At this polarized charge-form, DPAF moieties were not able to undergo further electron-oxidation process, consistent with the observed data. Furthermore, these CV results may be correlated with or indicative of similar charge-polarization phenomena to occur in the solid state of (*cis*-*cup*-**3**-C_9_)-encapsulated core-shell **5**-NPs.

The reflectivity of a subject is controlled by the refractive index of the subject surface medium that is a function of the product of permittivity (*ε*_r_) in a complex form and permeability (*µ*). Both are relevant material parameters in response to incident electromagnetic waves. The former complex permittivity can be presented by the equation *ε*_r_* = *ε*_r_′ − *iε*_r_″, where *ε*_r_′ is the real part as the relative dielectric constant and *ε*_r_″ is the imaginary part as the loss factor. The former parameter *ε*_r_′ is dependent on charge-polarization of the material at the wavelength of measurements. In the experimental data collection, the complex relative electric permittivity (and) values were measured in terms of complex reflection coefficient of electromagnetic waves in proportion to a complex scattering parameter, defined as S_11_. The scattering parameter S_11_ was measured by a high-performance coaxial probe (Agilent 85070E) [27]. It was then converted to complex relative electric permittivity (*ε*_r_*) values using the Nicolson-Ross algorithm [28]. From this measurement and calculation, the value of *ε*_r_′ was derived. 

Surface plasmon resonance (SPR) energy phenomena has been demonstrated and applied in many technological areas including the use of it as an alternative means to increase either light absorption or scattering in a thin film to enhance solar cells efficiency [29,30]. In principle, SPR energy occurs as the result of collective oscillation of surface electrons in gold nanoparticles that is induced by the interaction with the incident light. It leads to polarization with the formation of polaritons leading to the influence of absorption cross-section enhancement and light emitting [31]. In our study, we applied core-shell nanoparticles to investigated the possibility of tuning the SPR energy band to match with absorptions of dielectric materials for the induced intramolecular electron polarization that led to the modulation of dielectric constant and related physical property of the material layer on core-shell nanoparticles of **5**-NPs. The approach coincides with our recent results of ultrafast photoinduced intramolecular e^−^-transfer phenomena in femtoseconds [19,20]. Therefore, we found that a near-field effect within a few nanometers was crucial to enhance SPR energy accumulation during the energy-transfer event between plasmonic AuNPs and C_60_-(antenna)_x_ core-shell layers by direct contacts. Accordingly, we assembled a multiple layered structure of NPs having an inner magnetic *γ*-FeO_x_@AuNP core encapsulated by an outer *cis*-*cup*-tris[C_60_(>DPAF-C_9_)] shell, as described above. The NP fabrication was coupled by the control of the layer thickness of *cis*-*cup*-**3**-C_9_ in terms of the quantity weight ratio to that of *γ*-FeO_x_@AuNP (**4**-NPs). 

We first evaluated the degree difference of dielectric property amplification between **1**-C_9_ (giving **6**-NPs) and *cis*-*cup*-**3**-C_9_ (giving **5**-NPs) using the same plasmonic core-shell *γ*-FeO_x_@AuNP core. As a result, Figure 5B showed irradiation time-dependent relative dielectric constant (*ε*_r_′) curves of **5**-NPs and **6**-NPs taken at the microwave frequency of 1.0 GHz during and after a 60-min white LED light illumination (corresponding to a total light fluence of 12.8 J/cm^2^). Apparently, microwave EM wave showed a higher sensitivity to the NP surface materials for us to note the detected initial dielectric constant value at the time zero min being comparable in value to that of organic **1**-C_9_ (*ε*_r_′_0min_ = 1.55) and *cis*-*cup*-**3**-C_9_ (*ε*_r_′_0min_ = 2.48) even though the inner shell layer of AuNPs and the core *γ*-FeO_x_ NPs having a much higher *ε*_r_′_0min_ (=9.0) value. A slightly higher *ε*_r_′_0min_ for the latter is interesting since higher dielectric constant organics may be advantageous in energy devices. In all experiments, white light illumination led to a slight rise in temperature to 35–45 °C in the first 15 min. Slight increase of chamber temperatures away from 25 °C resulted in a small increase of the *ε*_r_′ value in a similar degree among these two samples. The *ε*_r_′ value was then remaining relatively steady during the rest of irradiation period up to 60 min. Upon turning-off the light at the end of 60-min irradiation, we observed a large sharp raise of the *ε*_r_′ value for both samples while the chamber temperature dropped quickly back to 25 °C. Sharp increase of the permittivity at the light-off stage was found to reach a peak maximum (*ε*_r_′_max_) value of 5.75 for **5**-NPs (Figure 5(Bb)) and 3.75 for **6**-NPs (Figure 5(Ba)). They can be accounted by a ratio of *ε*_r_′_max_/*ε*_r_′_0min_ in a 2.35- and 2.42-fold increase for **5**-NPs and **6**-NPS, respectively. Similar calculations based on the ratio of *ε*_r_′_max_/*ε*_r_′_60min_ (the *ε*_r_′ value at time 60-min) were found to be 1.76- and 1.74-fold increase in the *ε*_r_′ value for **5**-NPs (*ε*_r_′_60min_ = 3.26, Figure 5(Bb)) and **6**-NPs (*ε*_r_′_60min_ = 2.16, Figure 5(Ba)), respectively. Apparently, fabricated core-shell structural configurations were capable of inducing permittivity amplification at a RF-frequency of 1.0 GHz. 

**Figure 6:**
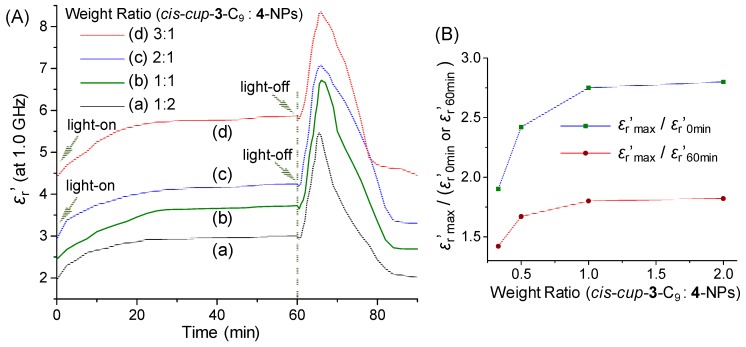
(**A**) Irradiation time-dependent relative dielectric constant (*ε*_r_′) amplification of trilayered core-shell nanoparticles of (*γ*-FeO_x_@AuNP)@{*cis*-*cup*-tris[C_60_(>DPAF-C_9_)]}_n_ (**5**-NPs) with a different weight ratio of *cis*-*cup*-**3**-C_9_ vs *γ*-FeO_x_@AuNP (**4**-NPs) at the frequency of 1.0 GHz with a white LED light irradiation period of 60 min. (**B**) Relative dielectric constant (*ε*_r_′) ratios of the values at peak maximum vs that at either 0 min or 60 min, showing a progressive increase of the degree of dielectric amplification.

We also investigated the weight ratio (*cis*-*cup*-**3**-C_9_/*γ*-FeO_x_@AuNP)-dependent dielectric property of trilayered (*γ*-FeO_x_@AuNP)@{*cis*-*cup*-tris[C_60_(>DPAF-C_9_)]}_n_ (**5**-NPs) for the comparison at the frequency of 1.0 GHz. Similar sharp increase of the permittivity at the light-off stage was found for all samples to reach a peak maximum (*ε*_r_′_max_) value of 5.46 (Figure 6(Aa)), 6.60 (Figure 6(Ab)), 7.03 (Figure 6(Ac)), and 8.39 (Figure 6(Ad)) for the weight ratio of 1:2, 1:1, 2:1, and 3:1, respectively. They can be accounted by a corresponding ratio of *ε*_r_′_max_/*ε*_r_′_0min_ in a 2.80- (*ε*_r_′_0min_ = 1.95), 2.75- (*ε*_r_′_0min_ = 2.40), 2.42- (*ε*_r_′_0min_ = 2.90), and 1.90-fold (*ε*_r_′_0min_ = 4.42) increase, respectively. Interestingly, a higher quantity of *cis*-*cup*-**3**-C_9_ applied led to a progressively increase of the initial dielectric *ε*_r_′_0min_ value up to 4.42 higher than that of many organic materials and polymers. This higher *ε*_r_′_0min_ value resulted in a lower degree of amplification *ε*_r_′_max_/*ε*_r_′_0min_. However, a high dielectric *ε*_r_′_max_ value of 8.39 (Figure 6(Ad)) achieved in organic-inorganic hybrid nanomaterials is significant for potential applications. Finally, similar calculations based on the ratio of *ε*_r_′_max_/*ε*_r_′_60min_ were found to be 1.82- (*ε*_r_′_60min_ = 3.0, Figure 6(Aa)), 1.80- (*ε*_r_′_60min_ = 3.67, Figure 6(Ab)), 1.67- (*ε*_r_′_60min_ = 4.21, Figure 6(Ac)), and 1.42-fold (*ε*_r_′_60min_ = 5.91, Figure 6(Ad)) increase, respectively. Relative dielectric constant ratios of either *ε*_r_′_max_/*ε*_r_′_0min_ or *ε*_r_′_max_/*ε*_r_′_60min_ as the *ε*_r_′ value shown at each peak maximum vs that at either 0 min or 60 min, respectively, in Figure 6B indicated a progressive increase of the degree of dielectric amplification upon the quantity increase of *cis*-*cup*-**3**-C_9_ up to a weight ratio of *cis*-*cup*-**3**-C_9_/*γ*-FeO_x_@AuNP as 2.0/1.0. 

## 3. Experimental Section

### 3.1. Chemicals and Reagents

Reagents of aluminum chloride (AlCl_3_), tris(dibenzylideneacetone)dipalladium(0) [Pd_2_(dba)_3_(0)], *α*-bromoacetyl bromide, *rac*-2,2′-bis(diphenylphosphino)-1,1′-binaphthyl (BINAP), sodium *t*-butoxide, and 1,8-diazabicyclo[5.4.0]undec-7-ene (DBU) were purchased from Aldrich Chemicals (St. Louis, MO, USA) and used without further purification. A C_60_ sample with a purity of 99.0% was purchased from Term USA, Inc. (Fort Bragg, CA, USA) The anhydrous grade solvent of THF was refluxed over sodium and benzophenone overnight and distilled under reduced pressure (10^−1^ mmHg). Sodium sulfate was used as the drying agent. The precursors 1,3,5-tris(*N*-phenylamino)benzene (TPAB) and 2-bromo-9,9-bis(3′,5′,5′-trimethyl-1′-hexyl)fluorene (BrF-C_9_) were synthesized according to our previous procedures [22]. 

### 3.2. Instruments for Spectroscopic Measurements 

Infrared spectra were recorded as KBr pellets on a Thermo Nicolet AVATAR 370 FTIR spectrometer (Thermo Scientific Nicolet, Waltham, MA, USA). UV-vis spectra were recorded on a PerkinElmer Lambda 750 UV spectrometer (PerkinElmer, Shelton, CT, USA). ^1^H NMR and ^13^C NMR spectra were recorded on Bruker & Spectrospin Avance 500 (Bruker, Billerica, MA, USA). The high performance liquid chromatography (HPLC) was performed using the µPorasil^TM^ 125 Å Column (10 µm, 35 × 300 mm, Waters, Milford, MA, USA) with a mixture of hexane-EtOAc as the mobile phase and the flow rate of 1.0 mL/min. The eluted compounds were detected by their UV absorption at *λ* 350 nm. 

Cyclic voltammetry (CV) was record on EG&G Princeton Applied Research 263A Potentiostat/Galvanostat (Ametek Inc., Berwyn, PA, USA) using Pt metal as the working electrode, Ag/AgCl as the reference electrode, and Pt wire as the counter electrode at a scan rate of 10 mV/s. The solution for CV measurements was prepared in a concentration of 1.0 × 10^−3^ M in CH_3_CN–CH_2_Cl_2_, containing the electrolyte Bu_4_N^+^-PF_6_^−^ (0.2 M). Transmission electron microscopy (TEM) measurements were carried out on Philips EM400T Transmission Electron Microscope (FEI Co., Hillsboro, OR, USA). In the sample preparation, a carbon-Copper film grid in a 200-mesh size was used as the supporting plate for directly coating of a nanoparticle sample in a solution of 1.0 × 10^−6^ M on the grid. All solvents were removed and dried by the freeze-dry technique to prevent the solvent-removal induced particle aggregation.

### 3.3. Synthesis of N^1^,N^3^,N^5^-Tris(9,9-di(3′,5′,5′-trimethyl-1′-hexyl)fluoren-2-yl)-1″,3″,5″-tris(phenylamino)benzene, tris(DPAF-C_9_) as **2**-C_9_

Synthetic procedure of tris(DPAF-C_9_) was followed by and slightly modified from the recently reported methods [22]. A typical procedure was given as follows. A mixture of BrF-C_9_ (10.0 g, 20.3 mmol, excess), TPAB (1.16 g, 3.30 mmol), Pd_2_(dba)_3_(0) (0.023 g, 0.25 mol%), BINAP (0.046 g, 0.75 mol%), and sodium *t*-butoxide (1.94 g, 20.3 mmol) taken in anhydrous toluene (75 mL) was heated to refluxing temperature under nitrogen for a period of 72 h. After being cooled to room temperature, the reaction mixture was washed with water for three times and dried over sodium sulfate. A crude brown colored paste was obtained after evaporating the solvent. It was subjected to column chromatography purification using silica gel as the stationary phase and hexane-ethylacetate (9:1) as the eluent. The product of tris(DPAF-C_9_) was collected at *R*_f_ = 0.8 as light yellow solids in 83% yield (4.35 g). 

Since the HPLC indicated a mixture of tris(DPAF-C_9_) stereoisomers, further separation and isolation were carried out by thin-layer chromatography (TLC), using silica gel as the stationary phase and hexane-ethylacetate (9.5:0.5, *v*/*v*) as the eluent. A mid-broad band at *R*_f_ 0.55−0.7 was identified as the tris(DPAF-C_9_) products that can be cut evenly into two parts at *R*_f_ 0.55–0.63 (as the bottom cut) and *R*_f_ 0.63–0.7 (as the top cut). Based on their corresponding ^1^H NMR spectra, the top cut was confirmed to be the pure *cis*-*cup*-tris(DPAF-C_9_) stereoisomer and the bottom cut was consisting of a mixture of *trans*-*chair*-tris(DPAF-C_9_) and *cis*-*cup*-tris(DPAF-C_9_) stereoisomers in the ratio of roughly 2:3. This fraction was subjected to further TLC separation and elution two more times using hexane-ethylacetate (9.7:0.3, *v*/*v*) as the eluent for the first run and the similar mixture (9.8:0.2, *v*/*v*) for the second run to obtain *cis*-*cup*-tris(DPAF-C_9_) in improved purity and the pure form of *trans*-*chair*-tris(DPAF-C_9_) fraction.

Spectroscopic data of *cis*-*cup*-tris(DPAF-C_9_): FT-IR (KBr) *υ*_max_ 3064 (w, aromatic C-H stretching), 3038 (w), 3011 (w), 2956 (vs, aliphatic C-H stretching), 2908 (m), 2865 (s), 1596 (m), 1584 (s, C=C), 1495 (s, anti-symmetric deformations of CH_3_ groups and scissor vibrations of CH_2_ groups), 1468 (m), 1450 (s), 1363 (m, symmetric deformations of CH_3_ groups), 1296 (m, asymmetric stretching vibrations of C-N-C), 1246 (m, asymmetric stretching vibrations of C-N-C), 1215 (w), 1176 (w), 1157 (w), 1030 (w), 933 (w), 826 (w), 754 (m), 736 (s, C-H out-of-plan deformation), 710 (m, C-H out-of-plan deformation), 694 (m), and 508 (w) cm^−1^; UV-vis (EtOAc, 1.0 × 10^−5^ M) *λ*_max_ (*ε*) 323 (5.62 × 10^4^ L mol^−1^ cm^−1^) and 353 nm (5.65 × 10^4^ L mol^−1^ cm^−1^); ^1^H NMR (500 MHz, CDCl_3_) *δ* 7.58 (s, 3H, br), 7.52 (d, 3H), 7.32–7.21 (m, 9H), 7.09–7.01 (m, 18H), 6.82 (t, 3H), 6.55 (s, 3H), 1.92–1.67 (m, 12H), 1.10 (m, 6H), and 0.97–0.39 (m, 96H); ^13^C NMR (500 MHz, CDCl_3_) *δ* 151.7, 150.0, 149.2, 147.7, 146.5, 141.2, 136.8, 129.2, 126.6, 126.6, 124.0, 122.9, 121.7, 120.0, 119.3, 115.6, 54.5, 51.0, 50.6, 50.6, 38.3, 38.1, 37.4, 37.6, 33.1, 32.7, 31.1, 30.2, 29.6, 29.4, 27.2, 22.9, and 22.4; MALDI–TOF MS calcd for C_117_H_153_N_3_, *m*/*z* 1600.21; found, *m*/*z* 1601.26 (MH^+^), 1602.30, 1603.29, 1260.95, 1092.81, 752.23 and 508.79 (PhN^+^H + fluorene-C_9_). 

Spectroscopic data of *trans*-*chair*-tris(DPAF-C_9_): FT-IR (KBr) *υ*_max_ 3062 (w, aromatic C-H stretching), 3037 (w), 3012 (w), 2955 (vs, aliphatic C-H stretching), 2925 (vs., aliphatic C-H stretching), 2855 (s), 1630 (vs), 1588 (s, C=C), 1495 (s, anti-symmetric deformations of CH_3_ groups and scissor vibrations of CH_2_ groups), 1450 (s), 1362 (m, symmetric deformations of CH_3_ groups), 1294 (m, asymmetric stretching vibrations of C-N-C), 1250 (m, asymmetric stretching vibrations of C-N-C), 1218 (w), 1158 (w), 1035 (w), 803 (w), 763 (m), 736 (m, C-H out-of-plan deformation), 709 (w, C-H out-of-plan deformation), 691 (m), and 510 (w) cm^−1^; UV-vis (EtOAc, 1.0 × 10^−5^ M) *λ*_max_ (*ε*) 322 (5.50 × 10^4^) and 353 nm (5.68 × 10^4^ L mol^−1^ cm^−1^); ^1^H NMR (500 MHz, CDCl_3_) *δ* 7.58 (s, 3H, br), 7.51 (d, 3H), 7.34–7.20 (m, 9H), 7.09–7.01 (m, 18H), 6.84 (m, 3H), 6.55 (m, 2H), 6.48 (m, 1H), 2.02–1.67 (m, 12H), 1.11 (m, 6H), and 0.97–0.39 (m, 96H); ^13^C NMR [similar to those of *cis*-*cup*-tris(DPAF-C_9_)]; MALDI−TOF MS calcd for C_117_H_153_N_3_, *m*/*z* 1600.21; found, *m*/*z* 1601.37 (MH^+^), 1602.36, 1603.35, 1604.40, 1260.00, 1093.86, and 508.73 (PhN^+^H + fluorene-C_9_).

### 3.4. Synthesis of N^1^,N^3^,N^5^-Tris(7-a-bromoacetyl-9,9-di(3′,5′,5′-trimethyl-1′-hexyl)fluoren-2-yl)-1″,3″,5″-tris(phenylamino)benzene, cis-cup-Tris(BrDPAF-C_9_)

To a suspension of AlCl_3_ (1.0 g, 7.5 mmol) in 1,2-dichloroethane (40 mL) at 0 °C was added *cis*-*cup*-tris(DPAF-C_9_) (0.7 g, 0.44 mmol) with vigorously stirring. The compound of *α*-bromoacetyl bromide (1.0 g, 5.0 mmol) was then slowly added over 10 min while maintaining the temperature between 0–10 °C. The mixture was warmed to ambient temperature and stirred overnight. The reaction was quenched by slow addition of water (50 mL) while maintaining the temperature below 45 °C. The organic layer was washed sequentially with dil. HCl (1.0 N, 50 mL) and water (50 mL × 2), and dried over sodium sulfate and then concentrated in vacuo to give the crude product as viscous yellow semi-solids. It was purified by column chromatography (silica gel) using hexane–EtOAc (9:1, *v*/*v*) as eluent to afford tris(Br-DPAF-C_9_) in 53% yield (0.46 g). Spectroscopic data: FT-IR (KBr) *υ*_max_ 3066 (w), 3035 (w), 2954 (s), 2907 (m), 2864 (m), 1733 (w), 1675 (s), 1594 (s), 1584 (s), 1492 (m), 1464 (m), 1429 (m), 1393 (w), 1364 (w), 1343 (w), 1288 (s), 1262 (m), 1205 (w), 1180 (m), 1107 (w), 1022 (w), 819 (m), 748 (m), 713 (m), and 696 (m) cm^−1^; UV-vis (EtOAc, 1.0 × 10^−5^ M) *λ*_max_ (*ε*) 304 (8.88 × 10^4^ L mol^−1^ cm^−1^) and 395 nm (1.10 × 10^5^ L mol^−1^ cm^−1^); ^1^H NMR (500 MHz, CDCl_3_) *δ* 7.94 (m, 3H), 7.88 (m, 3H), 7.63 (m, 3H), 7.56 (m, 3H), 7.22−6.92 (m, 21H), 6.56 (m, 3H), 4.46 (m, 6H), 2.11−1.67 (m, 12H), 1.10 (m, 6H), 0.99–0.26 (m, 96H). 

### 3.5. Synthesis of N^1^,N^3^,N^5^-Tris(7-(1,2-dihydro-1,2-methanofullerene[60]-61-carbonyl)-9,9-di(3′,5′,5′-trimethyl-1′-hexyl)fluoren-2-yl)-1″,3″,5″-tris(phenylamino)benzene, cis-cup-Tris[C_60_>(DPAF-C_9_)] as cis-cup-**3**-C_9_

A homogeneous solution of C_60_ (0.70 g, 0.97 mmol)) in anhydrous toluene (700 mL) was prepared by ultrasonication for a period of 1.0 h that was then stirred overnight under nitrogen. To this was added by *cis*-*cup*-tris(BrDPAF-C_9_) (0.40 g, 0.20 mmol) and 1,8-diazabicyclo[5.4.0]undec-7-ene (DBU, 0.21 g, 1.38 mmol) sequentially and stirred at room temperature for a period of 8.0 h. At the end of stirring, the reaction mixture was concentrated to a 10%-volume. Methanol (100 mL) was added to effect precipitation of the crude product, which was isolated by centrifugation. Further purification by column chromatography (silica gel) using a solvent mixture of hexane–toluene (3:2) as the eluent afforded *cis*-*cup*-tris[C_60_>(DPAF-C_9_)] as brown solids in 68% yield (0.52 g). Spectroscopic data: FT-IR (KBr) *υ*_max_ 2951 (m), 2923 (m), 2861 (w), 1681 (m), 1627 (s), 1594 (s), 1585 (s), 1490 (m), 1462 (m), 1429 (w), 1361 (w), 1317 (m), 1292 (m), 1247 (m), 1213 (m), 1184 (m), 1093 (w), 1035 (w), 908 (m), 819 (m), 732 (s), 698 (m), 575 (w), and 529 (s) cm^−1^; UV-vis (toluene, 1.0 × 10^−5^ M) *λ*_max_ (*ε*) 335 nm (1.76 × 10^5^ L mol^−1^ cm^−1^) and 402 nm (shoulder band, 4.19 × 10^4^ L mol^−1^ cm^−1^); ^1^H NMR (500 MHz, CDCl_3_) *δ* 8.46 (m, 3H), 8.30 (m, 3H), 7.81 (m, 3H), 7.63 (m, 3H), 7.31−6.70 (m, 21H), 6.60 (m, 3H), 5.67 (m, 3H), 1.92−1.67 (m, 12H), 1.14 (m, 6H), 0.70 (m, 96H); ^13^C NMR (500 MHz, CDCl_3_) *δ* 148.67, 148.56, 148.46, 147.89, 147.70, 147.18, 145.96, 145.82, 145.72, 145.68, 145.58, 145.47, 145.24, 145.13, 145.05, 144.73, 144.60, 144.48, 144.35, 144.28, 144.18, 144.14, 144.03, 143.94, 143.87, 143.72, 143.50, 143.41, 143.20, 142.92, 142.65, 142.50, 141.64, 141.35, 139.92, 137.07, 134.75, 134.03, 133.86, 129.61, 129.47, 124.34, 124.13, 123.53, 119.78, 116.61, 84.81, 72.88, 55.60, 51.55, 51.32, 51.02, 50.89, 45.07, 38.33, 38.02, 37.85, 37.67, 33.33, 31.31, 30.37, 29.93, 27.66, and 22.99. 

### 3.6. Preparation of cis-cup-Tris[C_60_>(DPAF-C_9_)]-Encapsulated γ-FeO_x_@AuNP Yielding Trilayered Core-Shell Nanoparticles, (γ-FeO_x_@AuNP)@{cis-cup-tris[C_60_(>DPAF-C_9_)]}_n_ (**5**-NPs)

Nanoparticles of *γ*-FeO_x_ and the corresponding plasmonic metallic gold-coated *γ*-FeO_x_@AuNP nanoparticles (**4**-NPs) were synthesized according to a slightly modified procedure reported previously [14,15]. A large scale preparation was carried out for the subsequent fabrication procedure using the same batch of materials to give consistency of *γ*-FeO_x_@AuNP characteristics in terms of the particle size and the Au-layer thickness. Spectroscopy data of **4**-NPs: FT-IR (KBr) *ν*_max_ 3027 (w), 2956 (w), 2923 (m), 2856 (m), 1631 (s), 1492 (m), 1461 (s), 1378 (m), 1328 (w), 1263 (w), 1166 (w), 1128 (w), 1076 (w), 1037 (w), 804 (m), 728 (m), and 588 (s) cm^−1^.

Encapsulation of *cis*-*cup*-tris[C_60_>(DPAF-C_9_)] on nanoparticles of γ-FeOx@AuNP was carried out by the method as follows. A mixture of *γ*-FeO_x_@AuNPs (100 mg) and *cis*-*cup*-**3**-C_9_ in a predefined weight ratio amount were dissolved in toluene (30 mL) with stirring for 30 min and then ultrasonicated for an additional >30 min until a clear solution being obtained showing a homogenized nanoparticles dispersion. The solution was concentrated via rotary evaporation to less than 3.0 mL in volume to increase the molecular contact of *cis*-*cup*-**3**-C_9_ to **4**-NPs. It was then diluted by toluene to a volume of 30 mL with stirring and subsequent sonication for 10 min to dissolve *cis*-*cup*-**3**-C_9_ fully. All encapsulated magnetic nanoparticles were physically removed from the container solution by the assistance of an external permanent magnet. The separated nanoparticles were washed repeatedly by ethanol and ether, followed by drying in vacuo to afford dark brown solids of **5**-NPs. Spectroscopy data: FT-IR (KBr) *ν*_max_ 3089 (w), 3064 (w), 3031 (w), 2952 (m, aromatic C-H stretching), 2927 (s, aliphatic C-H stretching), 2860 (m), 1679 (m, -C=O), 1636 (m), 1587 (s, -C=C-), 1492 (m), 1463 (m), 1430 (m), 1363 (w), 1291 (m, asymmetric stretching vibrations of C-N-C), 1247 (w), 1214 (m), 1187 (m), 1126 (w), 1091 (w), 1035 (m), 904 (m), 815 (w), 730 (m, C-H out-of-plan deformation), 695 (m), 576 (m), and 526 (s, C_60_>) cm^−1^. 

### 3.7. Measurements of Dielectric Properties and Permittivity as Relative Dielectric Constant (ε_r_′)

Microwave-responsive dielectric property measurements were carried out by the use of an Agilent Network Analyzer (Agilent Technologies, Inc., Santa Clara, CA, USA) equipped with an open-ended Agilent 85070E dielectric probe kit in a detection range of 200 MHz to 50 GHz. Prior to each measurement, the system base-line calibration was conducted by using open-ended, close-ended, and attenuated calibrators to ensure the minimization of signal instability that was induced by the cable connection and system drift errors. In the data collection, a complex scattering parameter, defined as S_11_, was measured and converted to relative complex electric dielectric constant values using Agilent 85071E Materials Measurement Software. This complex form of dielectric constant (ε*) consists of both a real (*ε*_r_′) and an imaginary (*ε*_r_″) part. The former term represents the value of dielectric constant and the latter is defined as the loss factor. 

All permittivity measurements were performed in a custom-built chamber that was conducted under a circumferentially uniform illumination environment. The uniformness was achieved by the installation of a reflective half-circular aluminum plate at the back-wall side surrounding the testing tube which was located at the center of the chamber. The light source used in this measurement included a collimated white LED light with an output power of 2.0 W (Prizmatix, Southfield, MI, USA). The light beam was allowed to pass through a small window opening at the front side of the chamber for the sample irradiation. Some reflected light beams were designed to refocus from the back-side aluminum mirror plate back to the sample-containing tube. We installed four small fans with two at the top and one at each side-wall of the chamber to control and prevent the temperature increase inside the chamber during the experiment. The illumination period was fixed at 60 min. Poly(dimethylsiloxane) (PDMS, 1.0 g) semi-solid was applied as a polymer matrix host which is capable of forming a paste-like material sample in mixing with a predefined quantity of nanoparticles. In a typical preparation, a mixture of PDMS and core-shell nanoparticle materials (100 mg) were blended by dissolving both components in ethyl acetate (20 mL) in a testing tube under sonication. It was followed by solvent evaporation to yield paste-like semi-solid materials filling in the tube.

## 4. Conclusions

We designed and synthesized novel 3D-configurated stereoisomers *cis*-*cup*-tris[C_60_>(DPAF-C_9_)] and *trans*-*chair*-tris[C_60_>(DPAF-C_9_)] in good yields. Efficient chromatographic separation was carried out to obtain both stereoisomers in a pure form with their corresponding nanostructure verified by spectroscopic techniques. The former with three C_60_> cages per molecule facing at the same side of the geometrical molecular cup-shape was proposed to provide excellent binding interaction forces at the gold surface of core-shell *γ*-FeO_x_@AuNP nanoparticles to direct the subsequent formation of a fullerene cage array (defined as *fullerosome*). Upon photoactivation of the Au-layer and *cis*-*cup*-tris[C_60_>(DPAF-C_9_)] itself, the level of photoinduced intramolecular e^−^-transfer from DPAF to C_60_> moieties was found to be largely enhanced by the accumulated plasmonic resonance energy at the near-field surface. Distribution of resulting negative charges along the outer (C_60_>)-derived fullerosome shell layer of the trilayered NPs was correlated with the detected photoswitchable dielectric amplification changes using white LED light at 1.0 GHz. This class of new materials exhibits potential in microwave applications as its photoswitching responsive wavelength range is being extended to cover WiFi-bands over 2.4/3.6/5.0 GHz.
